# The Spectral Game: leveraging Open Data and crowdsourcing for education

**DOI:** 10.1186/1758-2946-1-9

**Published:** 2009-06-26

**Authors:** Jean-Claude Bradley, Robert J Lancashire, Andrew SID Lang, Antony J Williams

**Affiliations:** 1 Drexel University, Department of Chemistry, 32nd and Chestnut Streets, Philadelphia, Pennsylvania 19104, USA; 2 Department of Chemistry, The University of the West Indies, Mona Campus, Kingston 7, Jamaica; 3 Oral Roberts University, Department of Computer Science and Mathematics, 7777 S. Lewis Ave, Tulsa, Oklahoma 74171, USA; 4 ChemZoo Inc, 904 Tamaras Circle, Wake Forest, North Carolina 27587, USA

## Abstract

We report on the implementation of the Spectral Game, a web-based game where players try to match molecules to various forms of interactive spectra including 1D/2D NMR, Mass Spectrometry and Infrared spectra. Each correct selection earns the player one point and play continues until the player supplies an incorrect answer. The game is usually played using a web browser interface, although a version has been developed in the virtual 3D environment of Second Life. Spectra uploaded as Open Data to ChemSpider in JCAMP-DX format are used for the problem sets together with structures extracted from the website. The spectra are displayed using JSpecView, an Open Source spectrum viewing applet which affords zooming and integration. The application of the game to the teaching of proton NMR spectroscopy in an undergraduate organic chemistry class and a 2D Spectrum Viewer are also presented.

## Background

Technology has made up to date information readily available to students through open course materials, recorded lectures, e-books, blogs, etc., and is offering additional options to distributing information directly to students other than through traditional lectures and textbooks[1]. As a consequence, an increasingly important role for educators is one where they become guides to knowledge and understanding; teaching skills and techniques, which now include information literacy skills – showing students how to locate, evaluate, and effectively use knowledge – and then to reinforce skills, knowledge and understanding through practice.

Traditional ways to reinforce skills, knowledge, and understanding through practice include homework, quizzes and labs. These traditional techniques can be enhanced with the use of freely available technologies, and in a world where the gaming market is beginning to outperform both music and films[2], some instructors are using technology and new freely available data to create games that catalyze learning and aid in the teaching of chemistry. Word puzzles have been devised to teach first year general chemistry[3], named organic reactions[4] and basic chemistry concepts[5]. Popular game shows such as Taboo[6], Jeopardy! [7,8] and Who Wants to be a Millionaire?[9] have been adapted for general chemistry review. Card games have been devised to teach carbohydrate chemistry[10], element symbols[11], functional groups[12] and organic reactions[13]. An adaptation of BINGO has been used to teach nomenclature[14]. A version of the Name Game facilitates student interaction while reviewing chemistry concepts[15]. There is even an organic chemistry game that can be played on a cell phone[16].

For spectroscopy, atomic absorption spectra can be practiced by moving virtual samples into a flame[17]. Infrared spectra interpretation has been turned into a game by a modification of checkers[18] or as a quiz[19]. Other board games used to teach general chemistry include Concentration[20], CHeMoVEr to learn about balancing chemical equations[21]. In addition to teaching applications, games can be used to solve chemistry problems. For example, Foldit has been used to leverage crowdsourcing to solve cases of protein folding[22]. Russell has extensively reviewed older chemistry games[23]. In this report we describe an additional game that can be played to help learn organic chemistry – a game that was not possible just a few years ago – The Spectral Game.

## The spectral game

The interpretation of spectra has always been an essential skill for mastering organic chemistry and many students struggle to grasp the nuances of various spectroscopy techniques. One of the problems is that traditional textbook assignments are pre-selected to provide simple examples that rarely deviate from what was learned in class. We believe this can be a disservice to students in that it does not prepare them for real-world structure elucidation challenges. With these limitations in mind we took advantage of the availability of the present perfect storm of internet technologies, online databases of Open structure and spectral data and flexible and intuitive tools for the viewing of spectral data to design a spectral game to assist in the teaching of spectroscopy in an entertaining yet educational manner.

### Implementation: The spectral game website

The Spectral Game[24] was created by bringing together Open Source spectral data, a spectrum viewing tool and appropriate work flows for delivering these in a gaming fashion. The Open Source spectral data identifiers and the properties and identifiers of the chemical structures which they represent are drawn from the ChemSpider database[25] using freely available web services provided by ChemSpider. These data are parsed and stored on the Spectral Game server. The game uses these data to display a series of spectra selected randomly one at a time, together with a number of chemical structure images. The challenge for the player is to select the chemical structure that matches the displayed spectrum. Both the spectrum and chemical structure images are pulled dynamically from the ChemSpider database using an embed functionality described below.

The ChemSpider database is an online database of over 21 million chemical structures, related molecular properties, links out to over 200 different data sources and, in relation to this game, is an environment where spectral data can be uploaded and hosted for the benefit of the community. At the time of writing there are more than 1400 Open Data spectral data sets on ChemSpider. The majority of these are proton and carbon-13 NMR spectra but there are also infrared, near infrared, UV-Visible and mass spectra. The community participates in the addition of spectral data content by uploading data in JCAMP-DX format, to the website[26]. Data have also been provided as collections by educational facilities (Pacific Lutheran[27]), software providers (ACD/Labs[28]) and national laboratories (NIST[29]). In order to facilitate the easy exchange of both structure and spectral data ChemSpider has provided an embed functionality[30] similar to that provided at many other websites such as YouTube. These Open Data spectra are imported into the game using this functionality thereby allowing Open Data spectra to be called for display inside a webpage using a simple Javascript call as shown below.

**Figure 1:**
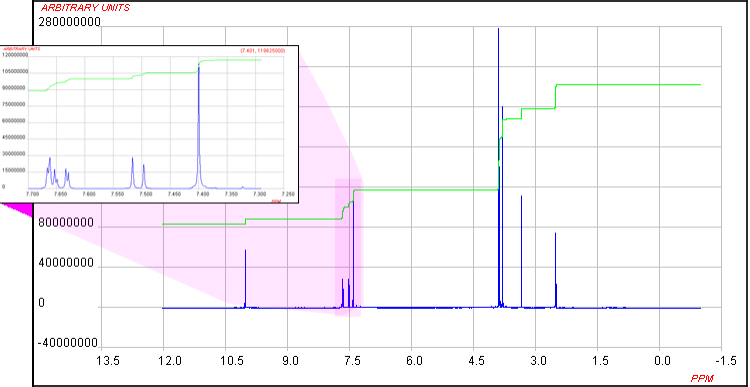
**Interactive spectrum viewer JSpecView**.

The javascript call of "<script type = "text/javascript" src=" http://www.chemspider.com/csjsapi.ashx?op=spec&tk=7283856c-a2d2-4634-8a11-1ea873f84244&bid=1317&w=750&h=400"></script>" calls the spectrum from ChemSpider and displays it in a webpage as is shown below in Figure 1.

The applet used to display the spectra (JSpecView[31]) was developed at UWI[32] and is now the primary Open Source spectrum viewing applet for displaying interactive spectra on the internet. It allows zooming, highlighting of regions within the spectrum, spectrum reversal and display of multiple spectra. Some changes were needed to the viewer code to allow for the suppression of sample details during the display so that users could not cheat by simply looking up the details of the material under study via the JCAMP-DX header. The first line of the header in a JCAMP-DX file is ##TITLE = and often contains the name of the sample. JSpecView has a menu option to display the header values and this would obviously make it very easy to answer the quiz. A new parameter was introduced (OBSCURE) that activates a routine to replace the value of the Title field with "unknown" so that any attempt to display the header lines would not give any advantage.

A brief introduction to the simplest JCAMP-DX protocol follows:

JCAMP-DX files consist of plain text that can be read and edited by simple text viewers. The simplest type is divided into two sections; the Header and the Data. The Header defines the type of spectrum, the source of the data, the instrument type and parameters as well as the dataset that follows in terms of start and end positions of the X values and deltaX. Many instruments make use of fixed separations of the X values and this is used in the method of compression for JCAMP-DX files. As mentioned above, the first line generally gives a description of the sample and looks like ##TITLE=, the second line is generally ##JCAMP-DX = with a version number. IR spectra are often found as version 4.14 while NMR need to be 5.01 since this corrected for problems of Shift References/Offsets not handled in earlier versions. JCAMP-DX version 6 that would cover 2D NMR was published as a draft for comment, but has not yet been finalised.

The Data section generally begins with ##DATAXY = (X++(Y..Y)) which is a shorthand notation for the idea that with fixed changes in the X values it is possible to put an X value at the start of the line and then provide a number of Y values with the understanding that these correspond to the next set of X values. The start of the next line begins again with an X value so that checks can be made to ensure that no line is missing or duplicated. A simple example from an IR file would be;

##XYDATA = (X++(Y..Y))

673 215867052 213571948 211374384 209305312 207332720 205205968 203152088

680 201167848 199108304 197021336 195063332 193056836 191013384 189185540

687 187358300 185164740 182889624 182557368 192119980 208444664 200564276

DeltaX was 1.0 and the Y values here have a ##YFACTOR = defined in the Header of ##YFACTOR = 9.313225746e-10 so that the actual values corresponding to X values of 673, 674, 675, 676 1/cm are calculated by multiplying by the YFACTOR to give:

0.198904 0.196858 0.194931 0.193094

A range of compression types exist to cram more Y values onto each line that make use of substituting the first digit by a letter etc.

The end of the file is indentified by ##END=

### Gameplay

**Figure 2:**
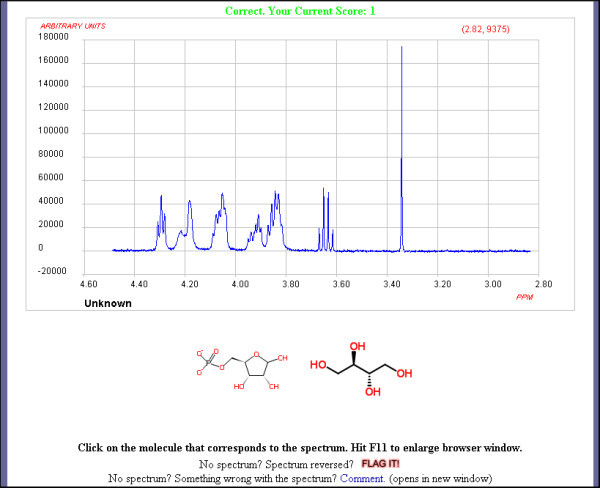
**An Early Round of the Spectral Game**.

At the beginning of the game two structures, one correct and one incorrect are shown below the spectrum, see Figure 2, and the user is asked to click on that structure that is the best match for the spectrum displayed.

**Figure 3:**
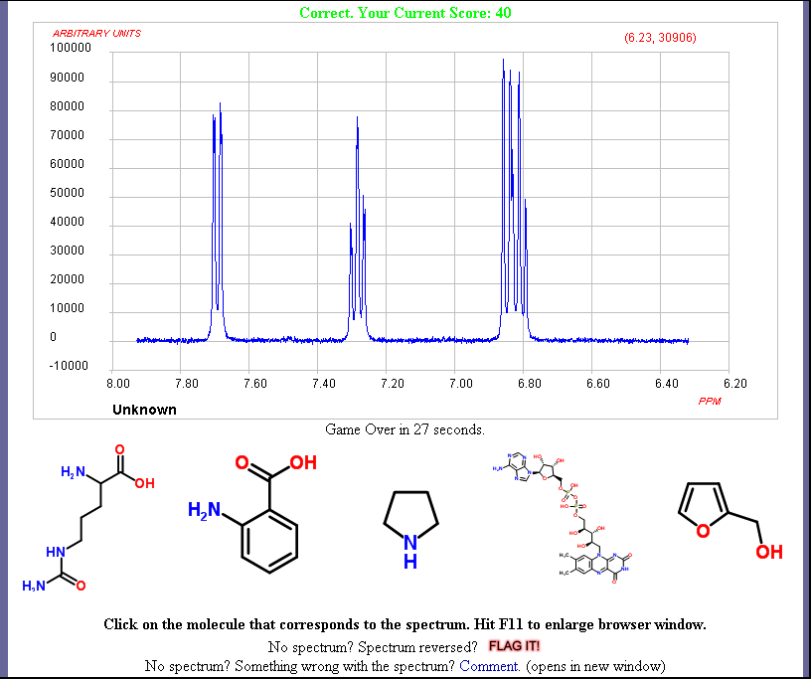
**Later rounds become timed and have more structures to choose from**.

The structures are extracted from ChemSpider using similar embedding functionality for chemical structures as used for spectra. If the user selects the correct structure then they proceed to the next set of spectrum and structures and the process is repeated. The player needs to examine the spectrum to compare various features to confirm or reject each of the structures. These include chemical shifts, multiplicities, peak intensity, functional groups and so on. In NMR users should be able to quickly distinguish aromatic protons from alkyl protons, aldehydic resonances from exchangeable carboxylic acid protons and methoxy singlets from methylene groups within a chain. In infrared spectra the user would be looking for specific functional group vibrations such as carbonyl groups. These simple filters can be enough to distinguish spectra-structure associations early in the game but complexity changes as the player progresses. The game becomes increasingly difficult with the number of associated structures increasing, to a maximum of five per spectrum. As the number of structures increase they also become more structurally similar. When a player reaches a score of forty, rounds also become timed, see Figure 3, and the player must select an answer before the countdown expires. The amount of time a player gets decreases as rounds progress to a minimum of ten seconds. So, even spectroscopy experts will find the game challenging.

Complexity also increases dramatically for the C-13 spectra where the number of carbon atoms in all of the structures is made equivalent to the number of carbon atoms present in the correct structure. This can be confusing until the player takes issues other than chemical shift into account: symmetry, peak intensity related to nature of carbon nucleus and so on.

The game continues until the player gets a spectrum validation wrong. At that point the player is given their performance relative to the list of both recent and top players. The game also allows players to associate with groups which allows for direct score comparison amongst members of the same group.

### Crowdsourced curation

**Figure 4:**
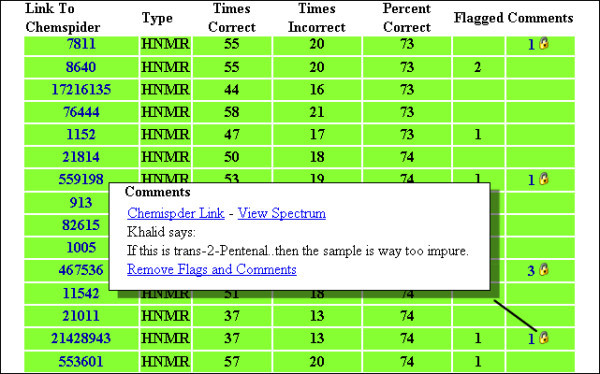
**Spectral curation interface**.

There may be a number of reasons that the player may get the spectrum assignment wrong. The user may simply not have the skills to perform the validation correctly and we have provided a wiki[33] of spectroscopy resources for players to brush up on their spectroscopy skills. Alternatively, the spectrum uploaded to ChemSpider itself may be wrong and incorrectly associated with a structure. One of the side benefits of the Spectral Game is the examination of the data and reporting of potential issues to the hosts of the game. As players progress through the game they can flag spectra initially displayed in reverse and leave comments associated with each of the spectra and curators on ChemSpider can review the data and take the appropriate actions. The percentage of time that a spectrum is matched correctly is also recorded giving a measure of how "difficult" the spectrum is to interpret. Website analytics show that within the last two months 3,434 unique visitors from 68 countries have combined to examine the spectra over 55,000 times as they played the game. Such crowdsourced curation efforts *have *resulted in the deletion or re-association of certain spectra from the database, have allowed the curators to re-reference the spectra or remove solvent peaks which were dominating the spectra to the point that compound resonances were not visible and have allowed the curators to add annotations to the spectra regarding the presence of impurities. An interface has been provided to ChemSpider curators to quickly review comments on individual spectra and link-through to ChemSpider to review, edit as appropriate and then remove the flags from the Spectral Game, see Figure 4. As a result of the Spectral Game, the quality of spectral data on ChemSpider has improved significantly with 3 spectra being removed from the database and over a dozen being processed to re-reference and remove solvent peaks. An additional side benefit is that the game has encouraged additional deposition of spectral data to the ChemSpider database thereby benefiting the chemistry community as a whole, not just the players of the Spectral Game.

### The spectral game in second life

**Figure 5:**
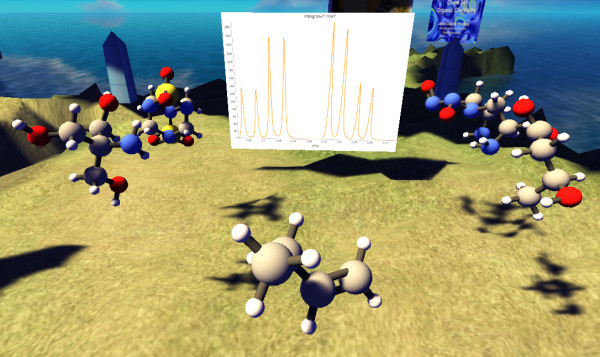
**The Spectral Game in Second Life**.

The Spectral Game was adapted to the multi-user virtual environment of Second Life by bringing together three tools[34]. First, we used the Orac molecule rezzer[35], a Second Life molecule building tool that takes SMILES, InChIs or InChiKeys and converts them to conformationally reasonable 3D structures. Second, we scripted a JCAMP spectrum viewer that allows interactive zooming and integration of desired regions via chat in Second Life. Finally, we used Open Data spectra from ChemSpider. The game begins by clicking on the spectrum display board. A spectrum then appears with several molecules in the surrounding area, see Figure 5. The player can zoom into any area of the spectrum by typing a command such as "zoom 1.2–2.5", which specifies the desired ppm range, in the chat box. By clicking on the correct molecule the player scores 2 points and gets another spectrum to analyze. Clicking on an incorrect molecule will cause the player to lose one point. When all spectra have been processed (a typical number is 5) the player is given their performance status in a list of top players. The game can also be terminated at any time by typing "quit."

### In class assessment

The Spectral Game was evaluated in one of the author's (JCB) undergraduate Organic Chemistry classes (CHEM242 at Drexel University) during the winter 2009 term[36]. Both the web and Second Life versions were used in different ways.

Workshops where the instructor led the class discussion while projecting the web version of the game were useful for a larger number of students, especially when students had just started learning to analyze spectra. Some IR and C NMR spectra were analyzed this way but the majority were H NMR. A class discussion would evolve about the key differences between the expected spectra of the molecules on display and then the instructor could zoom into relevant regions to explore those details. In this way, without any planning, all of the key concepts in the course relating to NMR were repeatedly reviewed. When the opportunities arose, simple coupling patterns, peak shifts, symmetry and diastereotopic groups were highlighted.

A main advantage of the Spectral Game compared to textbook problems is that real-world spectra were made available. Large solvent peaks (such as HOD at 4.8 ppm), peak distortions, overlapping peaks in complex coupling patterns and impurities were pointed out and the students were shown how to address this. Since the spectra are random, the instructor does not know ahead of time which spectra and molecules will be presented. This means that, on occasion, the instructor may not be able to solve the problems based on simple heuristics taught in class. This tended to happen more with carbohydrate derivatives. This was an opportunity to discuss other techniques that a working chemist might use in practice. We believe that such discussion is essential to help train students for real world research.

The game was also used individually by students. During class workshop time the instructor could circulate to assist students sequentially. Students were also encouraged to play the game from home by offering a prize for the top score during competition periods lasting about a week each. Two molecular model kits and a textbook were given out over the course of the term. Players can specify a group when logging in to play and this makes it easy to view the high scores within the class or compared to all the people from around the world playing. As noted by others, competition can be highly motivating for some students[10].

Because it requires more time to set up and demonstrate, the Second Life version was not used as much as the web version during the course. The ability to view molecules in 3D in Second Life is an advantage, especially for bridged cyclic structures. However, the main advantage of Second Life is the ability of students to interact with others in avatar form, whether it be other students in the class, the instructor or people from around the world[37]. This type of networking is not yet possible with the current web version of the game.

## 2D spectral game

Obtaining high quality 2D NMR open data sets is not as easy as finding 1D data. Fortunately, one of the authors (AJW) had been involved previously in a study involving automated versus human validation of 1D and 2D NMR data sets[38]. The paper describes a method for structure validation based on the simultaneous analysis of a 1D H NMR and 2D 1H-13C single-bond correlation spectrum such as HSQC or HMQC. When compared with the validation of a structure by a 1D HNMR spectrum alone, the advantage of including a 2DHSQC spectrum in structure validation is that it adds not only the information of 13C shifts, but also which proton shifts they are directly coupled to, and an indication of which methylene protons are diastereotopic. Using multiple real-life data sets of chemical structures and the corresponding 1D and 2D data, it was possible to unambiguously identify at least 90% of the correct structures in an automated fashion. ACD/Labs provided us with the 30 sets of 1D and 2D spectra presented in the publication together with the pairs of correct and incorrect structures. We used these data in a 2D NMR Spectral Game for users to start to learn how to use the combined information available from both 1D H1 and 2D HSQC data for identifying the correct structure. The development of this game has required the development of a new tool for visualizing 2D NMR spectra on the web (described below) and requires the player to use the H1 NMR spectrum in the standard JSpecView applet together with the 2D data display to interrogate the data and decide on the most appropriate match.

## Interactive 2D spectrum viewer

**Figure 6:**
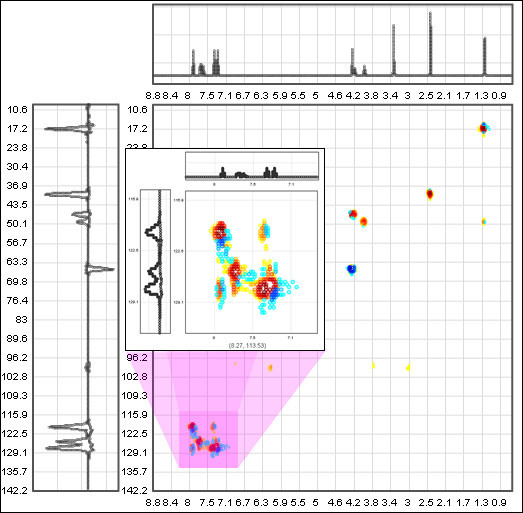
**Interactive 2D Spectrum Viewer**.

Multi-dimensional spectral data files lack a standardized format and are usually several megabytes in size. This poses problems for displaying interactive 2D spectral data on webpages. Lacking an Open Source viewer, websites usually display static images of spectral data. These issues resulted in us developing an Open Source interactive 2D spectrum viewer. The viewer is based on the Open Source flot[39] JavaScript library which allows for the interactivity – zooming of both the interior and side 1D spectra and updates of ppm values, see Figure 6. All this is achieved client side.

**Figure 7:**
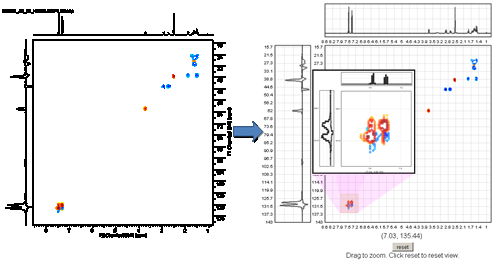
**The Same 2D Spectrum: Static PNG (left) and Interactive 2D Spectrum Viewer (right)**.

The data displayed in the 2D Spectrum Viewer is extracted from images of 2D spectral data using GD[40], a php graphics library. A php script downloads an image from a web server, in our case from ChemSpider, extracts the data pixel by pixel and exports the data in a format that can be read by the 2D Spectrum Viewer. The php code can be integrated with the viewer creating a dynamic 2D spectrum viewer that can be deployed in web pages, see Figure 7.

## Conclusion

New technology has great potential to benefit education. From this example, it should be clear how important Open Data can be for stimulating rapid re-mixing for educational examples. As more data become available, the usefulness of the Spectral Game and similar initiatives will become even greater.

## Availability and requirements

• Project name: Spectral Game

• Project home page: http://spectralgame.com

• Operating system: Web Based – Platform independent

• Programming languages: HTML, PHP, JavaScript, JAVA (JSpecView)

• Other requirements: Java 1.5

• License: MIT License (HTML, JavaScript) – GNU Lesser General Public License (JSpecView).

All files and related documentation are available from the project website http://spectralgame.com.

• Project name: 2D Spectrum Viewer

• Project home page: http://spectralgame.com/2d/2dviewer/

• Operating system: Web Based – Platform independent

• Programming languages: PHP, JavaScript

• Other requirements: GD, flot

• License: GNU Lesser General Public License.

## Competing interests

The authors declare that they have no competing interests.

## Authors' contributions

The Spectral Game was conceived by JCB and ASIDL. ASIDL wrote the code. AJW contributed spectra and structure images from ChemSpider and curates content. RJL modified JSpecView to remove title information from being viewable. JCB tested the game in an undergraduate organic chemistry class and provided prizes for contests involving gameplay. The 2D Spectral Game and 2D Spectrum Viewer was conceived by AJW and ASIDL. ASIDL wrote the code. AJW contributed 1D and 2D spectral sets and structure images from ChemSpider.
